# Validity of Sealant Retention as Surrogate for Caries Prevention – A Systematic Review

**DOI:** 10.1371/journal.pone.0077103

**Published:** 2013-10-23

**Authors:** Steffen Mickenautsch, Veerasamy Yengopal

**Affiliations:** Systematic Review initiative for Evidence-Based Minimum Intervention in Dentistry/Department of Community Dentistry, Faculty of Health Sciences, University of the Witwatersrand, Johannesburg, South Africa; University of North Carolina at Chapel Hill, United States of America

## Abstract

**Introduction/Aim:**

To appraise the clinical literature in determining whether loss of complete sealant retention as surrogate endpoint is directly associated with caries occurrence on sealed teeth as its clinical endpoint and to apply the appraised evidence in testing the null-hypothesis that the retention/caries ratio between different types of sealant materials (resin and glass-ionomer cement) is not statistically significant ( = Prentice criterion for surrogate endpoint validity).

**Methods:**

Databases searched PubMed/Medline, Directory of Open Access Journals; IndMed, Scielo. Systematic reviews were checked for suitable trials. The search terms: “fiss* AND seal*” and “fissure AND sealant” were used. Article selection criteria were: clinical trial reporting on the retention and caries occurrence of resin and/or glass-ionomer cement (GIC) fissure sealed permanent molar teeth; minimum 24-month follow-up period; systematic review or meta-analysis. Datasets and information were extracted from accepted trials. The principle outcome measure was the ratio of Risk of loss of complete retention to the Risk of caries occurrence per sealant type (RCR). Risk of bias was assessed in trials and sensitivity analysis with regard to potential confounding factors conducted. The null-hypothesis was tested by graphical and statistical methods.

**Results:**

The risk of loss of complete retention of sealant materials was associated with the risk of caries occurrence for resin but not for GIC based sealants. The difference between RCR values of the two sealant types was statistically significant (p<0.05). The null-hypothesis was rejected.

**Conclusions:**

The current clinical evidence suggests that complete retention of pit and fissure sealants may not be a valid surrogate endpoint for caries prevention as its clinical endpoint. Further research is required to corroborate the current results.

## Introduction

Sealing pits and fissures of teeth is an effective caries-preventive intervention [1]. It has been shown that up to 71% of occlusal decay is preventable after a single sealant application in a fissure [1]. Evidence regarding the efficacy and cost-effectiveness of sealants in reduction of occlusal carious lesions in molar teeth has been highlighted [2], [3]. Today the most commonly used sealant materials are resin- or GIC-based. Resin-based materials seal pits and fissures through micro-retention, created through tags after enamel acid etching [4]. Glass-ionomer cements (GIC) were introduced as alternative materials for sealing pits and fissures. In contrast to resin-based materials, GICs do not rely on micro-retention through tags after enamel acid etching, but on comparatively weaker chemical adhesion through ion exchange [5]. In contrast to resin and GIC- based sealants, only a few reports of clinical trials covering other types of sealant materials, such as compomers or ormocers, are available [6].

The first clinical evidence regarding the sealing of pits and fissures was based on splitmouth studies, which compared caries occurrence in sealed teeth and unsealed teeth [7]. Thus it was established that longevity of sealant material retention functions as a beneficial factor in the prevention of dental caries [8]–[10]. For the same reason, Kühnisch et al.; 2012 declared that, because optimum protection is guaranteed only if the sealant completely covers all pits and fissures, ‘intact sealant’ (as opposed to lost or partially retained sealant) is the leading fissure sealant criterion today [6]. Regression results have shown an association between retention rate and caries manifestation in support of this logical conjecture [7]. On the basis of the observation of ‘retention’ as a beneficial factor in the prevention of caries, the rate of sealant retention was forthwith assumed to be a valid surrogate endpoint for fissure sealant effectiveness.

A ‘surrogate endpoint’ has been defined as a measurement or physical sign, used as substitute for a ‘clinical endpoint’ [11]. A ‘true clinical endpoint’ has been defined as being a clinical, patient-relevant event of which the patient is aware, which the patient wants to avoid and which affects her/his quality of life [12]. Surrogate endpoints are commonly used in dental studies [13], as this offers several advantages above the use of clinical endpoints: surrogate endpoints make it easier to quantify and support trial precision and make the conduct of clinical trials simpler, shorter and consequently, less expensive [14].

For a surrogate endpoint to have meaning in relation to its clinical endpoint, it needs to be valid ( = Prentice criterion). The validity of any surrogate endpoint depends on its association with its true clinical endpoint and a surrogate should yield a valid test of the null-hypothesis (H0) of no association between type of treatment and treatment effect. This means that a surrogate variable will be valid only if: (i) the surrogate endpoint correlates with its true clinical endpoint and (ii) such correlation is independent of the type of treatment [15].

Therefore, complete sealant retention (or its loss) as a valid surrogate endpoint would need to fulfil the following two criteria:

- Direct association with caries absence (or caries occurrence) on sealed teeth;
- Independence of its ratio to caries ( = Retention/caries ratio or RCR) from type of sealant material used.

Thus, for the null-hypothesis to be accepted (H0: p>0.05), any difference in RCR values between sealants placed with different types of sealant materials should not be statistically significant.

Against this background, the objectives of this quantitative systematic review were to:

- Appraise the current clinical literature for evidence concerning the retention rate and caries occurrence on teeth sealed with the two most common sealant materials, resin and GIC;
- Establish whether, for both resin- and GIC-based sealants, loss of complete sealant retention as surrogate endpoint is directly associated with caries occurrence;
- Test the null-hypothesis that there are no statistically significant differences in the RCR values of sealants placed with resin or with GIC.

## Methods

The protocol of this systematic review has been published in an open access journal [16] and is freely available online (http://www.jmid.org). As the objective of this systematic review was methodological in nature and not focused on clinical outcomes (i.e. the effect size estimates between competing interventions were not investigated), the protocol was not eligible for registration with the International Prospective Register of Systematic Reviews (PROSPERO) [17].

### Systematic Literature Search

PubMed/Medline, Directory of Open Access Journals (DOAJ); IndMed and Scielo were searched by both authors (SM and VY), independently, using the search term: “fiss* AND seal*” (Table 1). For the string of search terms, the term “fissure AND sealant” was used in order to identify additional citations. The search period was limited to the publication period for/of the electronic database: up to 21.10.2012.

**Table 1:** Search strategy.

**Electronic database search**	Citations found*
DOAJ search strategy: 21.10.12 Online: http://www.doaj.org	
“fiss* AND seal*” (all fields)	43
“fissure AND sealant” (all fields)	1
**DOAJ/included total**	**0**
IndMed search strategy: 21.10.12 Online: http://indmed.nic.in/	
“fiss* AND seal*”	15
“fissure AND sealant”	0
**IndMed-search/included total**	**0**
PubMed search strategy: 21.10.12 Online: http://www.pubmed.org	
“fiss* AND seal*” (all fields)	3084
**Pubmed-search/included (Systematic review/meta-analyses reports)**	3**
**Pubmed-search/included (Clinical trials)**	**6**
Scielo search strategy: 21.10.12 Online: http://www.scielo.org/php/index.php	
“fiss* AND seal*” (integrated; regional)	20
“fissure AND sealant” (integrated; regional)	0
**Scielo-search/included total**	**0**
**Systematic review/meta-analysis reports (Reference check)	Citations found
**Kühnisch J, Mansmann U, Heinrich-Weltzien R, Hickel R. Longevity of materials for pit and fissure sealing-results** **from a meta-analysis. Dent Mater 2012; 28, 298–303.**	
Literature search in the MEDLINE, EMBASE and CENTRAL databases until 30.09.2011.	
Search terms: “fiss* AND seal*”	**95**
**Mickenautsch S, Yengopal V. Caries-preventive effect of glass ionomer and resin-based fissure sealants on** **permanent teeth: An update of systematic review evidence. BMC Res Notes 2011; 4∶22.**	
Literature search in the Biomed Central, Cochrane Oral Health Reviews, Cochrane Library,	
Directory Of Open Access Journals, Expanded Academic ASAP PLUS,	
Meta Register Of Controlled Trials, PubMed and Science-Direct databases until 26.08.2010.	
Search terms: “(GIC sealant* OR Glass ionomer cement sealant) AND (caries OR tooth decay)”	**1**
**Yengopal V, Mickenautsch S. Resin-modified glass-ionomer cements versus resin-based materials as fissure** **sealants - a meta-analysis of clinical trials. Eur Arch Paediatr Dent 2010; 11∶18–25.**	
Literature search in the Biomed Central, Cochrane Oral Health Reviews, Cochrane Library,	
Directory Of Open Access Journals, Expanded Academic ASAP PLUS,	
Meta Register Of Controlled Trials - mRCT, PubMed, Science-Direct, and 2 Lusophone databases:	
Bibliografia Brasileira Em Odontologia – BBO, Literatura Latino-Americana	
E Caribenha Em Ciências Da Saúde – LILACS databases until 15.04.2009.	
Search terms: “Pit and Fissure Sealants’[Mesh] and ‘Glass Ionomer Cements’[Mesh]”;	
“resin modified glass ionomer”; “fissure sealant*”; “‘selante’ [palavras]”;	
“‘cimentos de ionomeros de vidro’ [palavras]”; “‘CARIE’ [Palavras]’”.	**1**
**Total citations of clinical trials included**	**103**

* Duplications of trial citations excluded.

Citations were eligible for possible inclusion if in line with the following inclusion criteria:

- Clinical trial reporting on both the retention and the caries occurrence in resin and/or glass-ionomer cement (GIC) fissures of sealed permanent molar teeth (no distinction was made between different types of resin-based sealants or conventional and resin-modified GIC);
- Minimum 24-month follow-up period;
- Systematic review or meta-analysis reports on the topic.

Trial participants included all patients of any age, gender or place of origin.

Articles were further excluded if: premolars were included in the study and their data analysed with that of molar teeth; no caries was assessed; no computable data was reported; not all sealed teeth that were evaluated for sealant retention were also evaluated for caries. Articles that could not be traced in full copy were also excluded.

Electronic databases were searched for systematic review/meta-analysis reports, first, and reference lists of identified systematic review or meta-analysis reports were checked for suitable trials. Electronic databases were subsequently searched for additional clinical trials that were not included in the identified systematic review/meta-analysis reports using the same search terms.

Two reviewers (SM and VY) scanned titles and abstracts of identified citations from data sources in duplication. Articles with suitable titles but lacking listed abstracts were retrieved in full copy. All included articles were judged separately by the authors; for possible exclusion, with reason, or for acceptance, in line with the exclusion criteria. Disagreements between authors were resolved through discussion and consensus.

### Assessment of Internal Validity/Bias Risk

The risk of selection-, detection/performance- and attrition bias was assessed for controlled trials using published criteria [16]. Risk of attrition bias was assessed for uncontrolled, single arm trials or trials that compared either resin- or GIC-based sealants with other controls. The two reviewers conducted the assessment separately. Disagreements were resolved through discussion and consensus.

The assessment of publication bias risk by use of funnel plot and regression analysis is usually based on effect size estimates in relation to sample size. As the appraisal of effect size estimates between competing interventions formed no part of this systematic review, the assessment of risk of publication bias was not included.

### Data Extraction and Statistical Analysis

The principal outcome measure was the ratio of Risk of loss of complete retention to the Risk of caries occurrence on formerly sealed teeth per sealant type (RCR).

Both authors extracted data from the accepted articles independently, without being blinded to authors, institutions, journal names and trial results. The extracted data included (per type of sealant material at the end of each follow-up period):

N = Number of evaluated sealed teeth (N);
n_R_ = Number of teeth without completely retained fissure sealants/loss of complete material retention;
n_C_ = Number of sealed teeth with carious lesion/cavities.

The choice of using ‘loss of complete material retention’ was based on the consideration that “intact sealant”; i.e., complete sealant without material loss, has been adopted as the current leading fissure sealant criterion and is widely regarded as a surrogate for clinical success [6]. For the purpose of analysis, the extracted data (N; n_R_ and n_C_), per study, sealant type and follow-up period, was regarded as one single dataset (DS).

From the extracted data the following Risks were calculated:

R_R_ = Risk of loss of complete retention as the ratio (n_R_/N) of the number of teeth without completely retained sealant material (n_R_) to the number of evaluated sealed teeth (N) - considered as the ‘surrogate endpoint’.
R_C_ = Risk of caries occurrence as the ratio (n_C_/N) of the number of teeth with carious lesion/cavity development on formerly sealed teeth (n_C_) to the number of evaluated sealed teeth (N) - considered as being the ‘clinical endpoint’.

Simple linear regression analysis (SAS Institute Inc., SAS Software, version 9.3 for Windows, Cary, NC, USA: SAS Institute Inc., 2002–2010) was used to establish whether loss of complete sealant retention as surrogate endpoint is directly associated with caries occurrence for both, resin- and GIC-based, sealants. For this purpose a log transformation was utilized, to meet assumptions of linearity by computing the natural logarithm (ln) of the risks, R_R_ and R_C_. During regression analysis, ln(R_C_) formed the dependent variable and ln(R_R_) the independent variable. Trials were weighted in proportion to the number of investigated unique sealed teeth (Table 2). Where repeated measurements were taken per trial, the weight was based on the largest number of teeth. The analysis was conducted with clustering for repeated observations and/or multiple treatments within a trial. To determine direct association between R_R_ and R_C_, the slope of the regression line and the estimate of R_C_ at R_R = _0.002 (the smallest value of R_R_ and R_C_ in the data set) were assessed. For direct association, the slope should not differ significantly from 1, and the confidence interval for the estimate of R_C_ at R_R = _0.002 should include 0.002. The null-hypothesis that there are no statistically significant differences in the RCR values of placed resin or GIC sealants placed was statistically tested.

**Table 2:** Data subsets for analysis.

Data Subset	N	Nu	DS	A	B
Mat 1 - main	34 758	17 377	115	54	83
Mat 2 - main	6 424	3 768	35	22	24
Mat 1 - sens	3 154	3 154	18	14	18
Mat 2 - sens	2 307	2 307	14	14	14

N = Total number of sealed teeth in trials; Nu = Number of unique sealed teeth; DS = Number of datasets; A = Number of separate studies (clustering repeated observation points and/or testing of more than one material in a study); B = number of separate studies (clustering repeated observation points in a trial, only). Mat 1 = Resin based sealant; Mat 2 = Glass-ionomer based sealant; main = Main analysis result; sens = Sensitivity analysis result.

Testing was conducted through the following steps:

Calculation of RCR values, being the R_R_/R_C_ – ratios for both types of sealant per dataset (rounded to the second decimal);
Computing of the mean RCR with Standard Deviation (SD) from all datasets per sealant type;
Testing of differences between the mean RCRs of the two sealant types for statistical significance, using t-test on ln(RCR) to ensure normality of the data. (SAS Institute Inc., SAS Software, version 9.3 for Windows, Cary, NC, USA: SAS Institute Inc., 2002–2010).

The alpha level for statistical significance was set at 5%. Acceptance of the null-hypothesis was conditional upon the basis of lack of statistical significance (p>0.05) between the mean RCRs of resin- and GIC-based sealants.

Sensitivity analysis was conducted, with regard to:

Any influence that naïve-indirect comparison of unrelated data from uncontrolled studies might have had on the obtained results;
Inclusion of the filling and extraction components in the caries assessment;
Data independence with regard to different follow-up periods for the same sealed teeth that were presented in the same trial report.

As this systematic review did not include the pooling of results from accepted trials using meta-analysis, investigation of in-between-trial heterogeneity was not conducted.

Compliance of ‘loss of complete retention (R_R_)’ as surrogate endpoint with the Prentice criterion [15] was graphically assessed, following the methodology described by Baker and Kramer, 2003 [18]. For this purpose the following mean Risk values were computed from all datasets for each sealant type:

Mean R_R_ = Mean risk with standard deviation (SD) for loss of complete retention (R_R_);
Mean R_C_ = Mean risk with standard deviation (SD) for risk of caries occurrence on sealed teeth (R_C_).

The Baker-Kramer graph assumes perfect correlation between the two mean Risk values and places individual data points on straight lines (R^2^ = 1.00). The intercept of the line is set as zero because the surrogate endpoint is assumed to be proportional to the clinical endpoint. Compliance with the Prentice criterion [15] implies that the lines of both sealant types coincide on the graph [18].

## Results

### Systematic Literature Search and Data Selection

**Figure 1:**
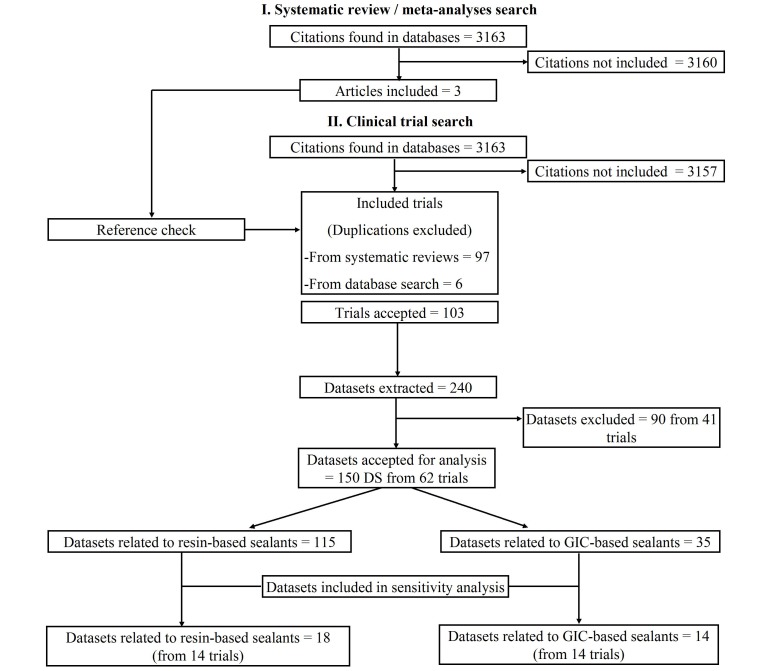
Flow diagram of trial selection.

Figure 1 provides information on the number of articles identified. Details of the search strategy, including search words and dates of electronic database search are shown in Table 1. The literature search of the electronic databases generated 3163 citations. Of these, six clinical trials and three systematic reviews/meta-analyses [6], [19], [20] were provisionally included. From the systematic reviews/meta-analyses 97 clinical trials were identified. Thus, a total of 103 trials were accepted for this review. Details of trial references, study design and length of observation period per trial are shown in File S1.

From the 103 trials, 240 datasets (DS) with relevance to sealant retention were extracted. From these, 90 datasets from 41 trials were excluded for the following reasons: exclusive investigation of molar teeth not made explicit (15 DS); caries assessment missing (35 DS); full article could not be traced (21 DS); no computable data reported (3 DS); not all sealed teeth that were evaluated for sealant retention were also evaluated for caries (16 DS). In total, 150 datasets from 62 trial reports were accepted for statistical analysis (File S1).

### Assessment of Internal Trial Validity/Bias Risk

Assessment of the internal validity of the 62 accepted trial reports was based on published criteria [16]. Details of the assessment results are presented in File S2. Of these, 18 trials were clinical controlled trials that directly compared resin- based and GIC-based fissure sealants; 39 trials were clinical controlled trials that compared either resin- or GIC based sealants with other controls and five trials were non-controlled longitudinal studies.

None of the 18 clinical controlled trials that directly compared resin-based and GIC-based fissure sealants included appropriate randomisation in their study methodology. Owing to the obvious difference in appearance between the two sealant types, no blinding was included. Loss-to-follow-up above 10% of the baseline sample size for both interventions was observed in 12 trials (Score 0); no loss-to-follow-up (Score A) was reported in three trials and one trial reported no loss-to-follow-up after two years and >10% after four years. Two trials reported loss-to-follow-up of <10% (Score B).

Loss-to-follow-up of >10% was reported in 37 of the other 44 trials (File S2).

### Extracted Data and Analysis

Of the accepted 150 datasets, 115 DS were related to resin-based, and 35 DS to GIC-based, fissure sealants. For the purpose of this study no distinction was made between molar tooth- or side-specific data.

#### Regression analysis

The results of the regression analysis are shown in Table 3. The adjusted R^2^ was low for all data subsets, i.e. only a low proportion of the variation in caries occurrence, ln(R_C_) was explained by the loss of complete sealant retention, ln(R_R_). A direct association between these variables could be demonstrated for resin based fissure sealants. In contrast, no association was found for GIC based sealants.

**Table 3:** Regression analysis results.

DataSubset	DS	AdjR^2^	Intercept						ln(R_R_)						Estimate of ln(R_C_) at ln(R_R_) = −6.215 (R_R_ = 0.002)							
			E	SE	t	p	95% CI		E	SE	t	p	95% CI		E	SE	DF	t	p	alpha	95% CI	
Mat 1 - main	115	0.28	−1.65	0.27	−6.07	<.0001	−2.19	−1.10	0.89	0.17	5.32	<.0001	0.56	1.23	−7.19	0.92	53	−7.83	<.0001	0.05	−9.031	−5.346
Mat 2 - main	35	0.099	−2.28	0.20	−11.28	<.0001	−2.70	−1.86	0.81	0.42	1.91	0.070	−0.07	1.68	−7.30	2.58	21	−2.83	0.010	0.05	−12.663	−1.928
Mat 1 - sens	18	0.23	−1.83	0.46	−3.95	0.0017	−2.83	−0.83	0.53	0.37	1.43	0.17	−0.27	1.32	−5.11	1.92	13	−2.66	0.020	0.05	−9.268	−0.960
Mat 2 - sens	14	0.22	−2.09	0.37	−5.66	<.0001	−2.88	−1.29	1.44	0.34	4.22	0.0010	0.71	2.18	−11.06	2.05	13	−5.39	0.0001	0.05	−15.484	−6.629

DS = Number of datasets; Mat 1 = Resin based sealant; Mat 2 = Glass-ionomer based sealant; main = Main analysis result; sens = Sensitivity analysis result; E = Estimate; SE = Standard error; CI = Confidence interval; DF = Degree of freedom; Adj = Adjusted.

#### Null-hypothesis testing

The mean RCR values were 9.64 (SD = 24.58) and 13.68 (SD = 13.72) for resin- and GIC-based fissures sealants, respectively. The difference was statistically significant (p = 0.017).

The results show that the ratio of the risk of loss of complete retention of sealant materials in pits and fissures to the risk of caries occurrence, was not sealant material independent, thus indicating non-compliance with the Prentice criterion.

#### Baker/Kramer plot

**Figure 2:**
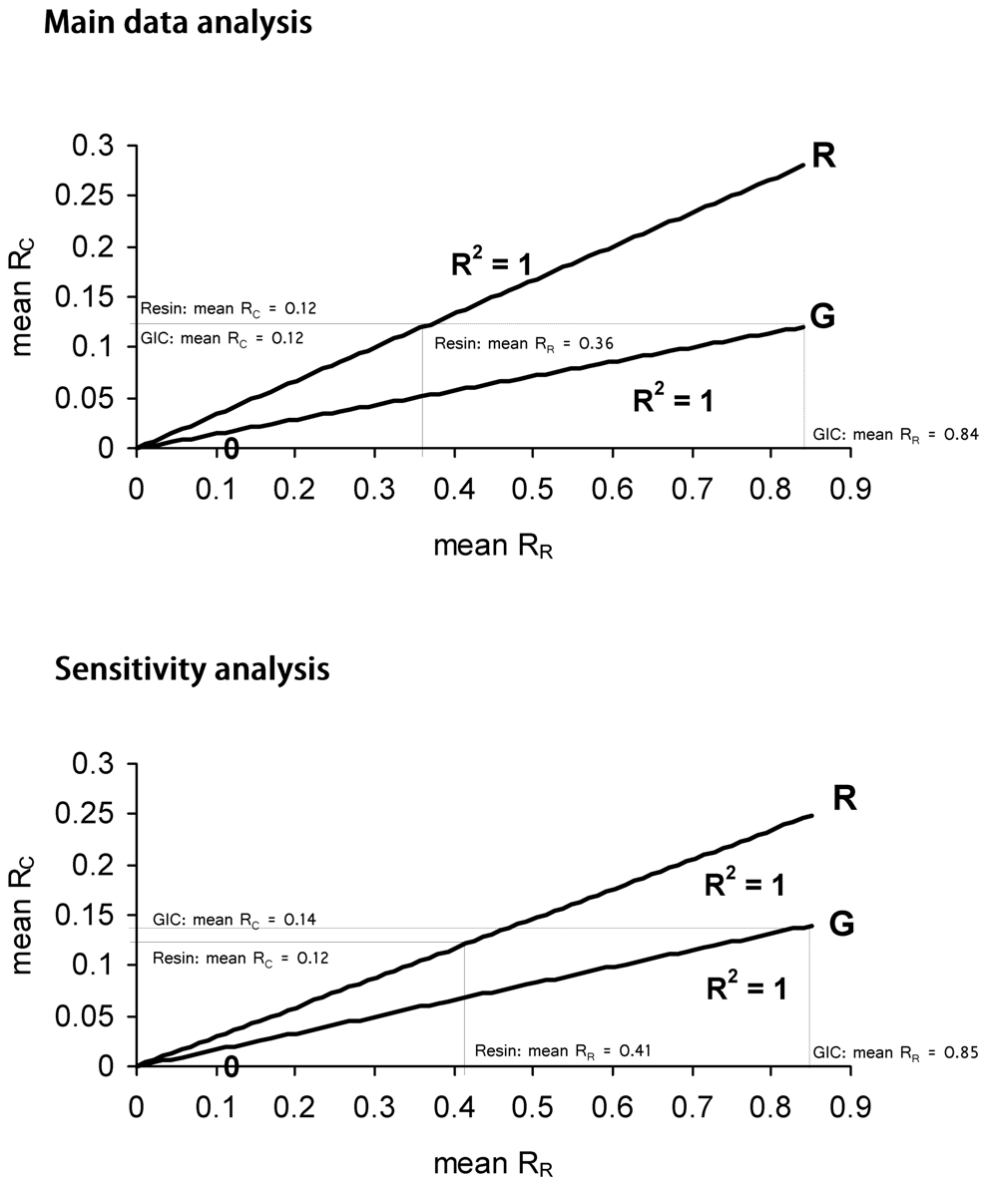
Graphical investigation of Prentice criterion compliance. R_C_ = Risk of carious lesion/cavity development; R_R_ = Risk of loss of complete sealant retention; GIC = Glass-ionomer cement based fissure sealants; Resin = Resin-based fissure sealants.

The mean risk of losing complete retention (R_R_) was 0.36 (SD = 0.23) and 0.84 (SD = 0.19) for resin- and GIC-based fissure sealants, respectively, indicating a risk more than twice as high for GIC as for resin. The mean risk of caries occurrence (R_C_) was 0.12 (SD = 0.14) and 0.12 (SD = 0.08) for resin- and GIC-based fissures sealant, respectively, indicating a similar risk for GIC and for resin. The graphical assessment of the mean values is shown in Figure 2.

#### Sensitivity analysis

Although inclusion of all eligible studies will have ensured the highest possible sample size of the analysed data, a large amount of this data was derived from uncontrolled and unrelated studies. This may have included an element of naïve-indirect comparison between resin- and GIC-based fissure sealants, with consequent risk of inflating the true results [21]. For that reason sensitivity analysis was conducted, by including only that part of the total study cohort that investigated retention and caries related to both types of sealant material. In addition, some of the recorded caries data was derived from DMF- filling and extraction components and some datasets concerning the same investigated sealed teeth but of different length of follow-up period were extracted from the same trial reports. Thus, only datasets of the longest per trial follow-up periods were included, only, and datasets derived from DMF- filling and extraction components datasets were excluded, due to uncertainty as to whether teeth were filled and/or extracted for caries reasons (File S3).

In total, 14 trials for each sealant type, with 32 (Resin = 18; GIC = 14) datasets were included for sensitivity analysis. The following results were established:

- In contrast to the main analysis results, simple linear regression analysis revealed no direct association between ln(R_C_) and ln(R_R_) for resin based fissure sealants. However, a direct association was found for GIC based sealants. For both materials, the adjusted R^2^ was low. Thus only a low proportion of the variation in caries occurrence, ln(R_C_) was explained by the loss of complete sealant retention, ln(R_R_) (Table 3).
- The mean risk of losing complete retention (R_R_) was 0.41 (SD = 0.28) and 0.85 (SD = 0.18) for resin- and GIC-based fissure sealants, respectively. The mean risk of caries occurrence (R_C_) was 0.12 (SD = 0.13) and 0.14 (SD = 0.10) for resin- and GIC-based fissure sealants, respectively;
- The mean values of the RCR ratios were 5.34 (SD = 7.35) and 14.61 (SD = 17.33) for resin- and GIC-based fissure sealants, respectively;
- The difference between the RCR ratios was not statistically significant (p = 0.069).

## Discussion

### Limitations of the Systematic Review Method

The aim of this review was not to seek validation of loss of complete retention of fissure sealant material as the potential surrogate endpoint in clinical trials. It therefore did not adopt recommendations suggested for this purpose [22]–[24]. Instead, simple linear regression, t-test, as well as recommended graphical methods [18] were applied as sufficiently appropriate methods.

Datasets concerning fissure sealants placed in premolar teeth and observation periods of less than 24 months were excluded. Although such exclusion is in line with investigations by other authors [6], [7], it may have limited the data available on this topic. Moreover, this study focused on resin- and GIC-based fissure sealant materials only and did not include ‘compomer’ and ‘ormocer’ materials. This decision was due to scarcity of available data regarding these other material types [6]. A further nine and seven datasets related to resin- and GIC sealants, respectively were excluded, as a potential risk of attrition bias was caused when some sealed teeth that were evaluated for retention loss were not evaluated for caries, as well. No distinction was made between different types of resin materials or between conventional and resin-modified GIC, as it was assumed that, in line with the current consensus concerning the validity of sealant retention as surrogate for caries, any differences in the retention rates would translate to similar differences in caries experience, regardless of the type of fissure sealant material used. Furthermore, factors such as ‘patient age’ or ‘length of observation period’ were not considered. The choice of “intact sealant” as the “leading fissure sealant criterion today” [6] naturally emphasises the sole validity of ‘retention’ as pivotal and true under any type of condition, regardless of factors such as ‘patient age’ or ‘length of observation period’.

### Assessment of Internal Trial Validity/Bias Risk

All of the accepted controlled trials appear limited by risk of either selection- and/or detection−/performance bias.

All of the 18 controlled trials that directly compared resin- with GIC sealants failed to report not only on evidence of successful sequence allocation and allocation concealment results, but also on necessary details about how sequence allocation and allocation concealment were attempted (File S2: assessment scores 0 and D) [16]. None of the trials, therefore, provide any guarantee that each patient had an equal chance of being allocated to either treatment group. Such potential selection bias may have caused more patients with, for example, higher caries risk to be allocated to one sealant group than to the other and thus artificially inflated the risk of caries occurrence in relation to risk of sealant retention loss.

From the onset, in all trials successful blinding or masking appeared not to have been possible, owing to the obvious differences in clinical appearance between GIC and resin sealants. For that reason, the allocation to either treatment group was visible to patients, operators and evaluators. However, difficulties in successful blinding still carry the danger of detection−/performance bias, which may thus have affected the trial results. Potential knowledge of superiority claims prior to the trial may have led evaluators to apply different rigor in their assessments regarding the two types of sealant. This in turn may have affected the primary outcome measure of this review (RCR) by artificially increasing one risk value above the other (R_R_ in relation to R_C_ or vice versa).

Most trials reported a lost-to-follow-up of >10% for either type of sealant (File S2). Such loss of data may have especially affected the presented regression results, particularly for the GIC-based sealant group, in terms of regression coefficient, standard error and R^2^-values.

Owing to these limits in internal validity, the evidence provided by the current data should be regarded with caution and requires corroboration through future updates of this systematic review.

### Statistical and Graphical Analysis

The regression results presented indicate a significant correlation of the surrogate with its clinical endpoint for resin- but not for GIC based sealants. Despite differences in regression method, the result for resin-based sealants is in agreement with previously observed regression outcomes [7] and indicates that complete material retention is associated with the caries-preventive efficacy. However, this appears not to be the case for GIC based sealants. The implication is that complete sealant retention as surrogate for caries prevention does not comply with the first condition of the Prentice criterion [15].

The lack of direct association between sealant retention and caries prevention, for GIC sealants may be explained by the fact that remnants of the GIC sealant material remain at the bottom of pits and fissures, even when the sealant is clinically judged as having been lost and thus continues to provide a caries preventive effect [25], [26]. Such remnants are retained because GICs have been observed to fracture cohesively and not adhesively like resin-based sealants [27]. In addition, glass ionomers have been found to continue the release of fluoride on a long-term basis [28]. The continued fluoride release from small amounts of GIC sealant material that remains at the bottom of pits and fissures may explain the lack of direct association between clinically identified loss of sealant retention and caries.

It is a common misconception that mere correlation suffices for a chosen endpoint to be a valid surrogate of its clinical endpoint [12]. In addition to such correlation, the Prentice criterion requires potential surrogate endpoints to also be able to capture the full net effect of the clinical outcome [15]. The loss of complete retention of sealant materials as surrogate endpoint does not fulfil this criterion. The regression results indicate no perfect correlation for either sealant material. Nonetheless, perfect correlation between the risk of loss of complete retention of sealant materials (R_R_ - horizontal axis) and risk of caries occurrence (R_C_ - vertical axis) for both material types was assumed in the Baker-Kramer graph, shown in Figure 2, by placing individual data points on straight lines (R^2^ = 1.00). As the clinical endpoint being assumed to be proportional to the surrogate endpoint, the intercept of both lines (G = GIC; R = Resin) is zero [18]. However, compliance with the Prentice criterion implies that both lines would coincide; i.e. a decrease in the mean risk of loss of complete retention of sealant materials (R_R_) would always translate to a similar decrease in the risk of caries occurrence (R_C_), regardless of type of material. In such case, correct inference about the clinical endpoint would always be obtained on the basis of the surrogate endpoint [15], [18]. This is not the case in loss of complete retention in relation to caries occurrence. Figure 2 clearly shows a difference in slope between the R and G lines.

When the retention/caries relationship was expressed in the form of the RCR-ratio, the difference between the two types of material was shown to be statistically significant (p<0.017).

### Sensitivity Analysis

The results of the sensitivity analysis were not consistent with the results of the main analysis: (i) a direct association between sealant retention and tooth caries was not observed for resin- based sealants but instead for GIC based sealants; (ii) the difference between the RCR ratios of resin- and GIC sealants was not statistically significant (p = 0.069). Such differences may be due to the confounding effects of comparing RCR values between unrelated clinical trials ( = naïve-indirect comparison); the possibility that some of that formerly sealed teeth were not filled and/or extracted because of caries; some form of chronological bias that may occur when results from different follow-up periods of the same fissure sealants are combined; or due to unknown factors. The exclusion of all affected datasets from the sensitivity analysis also reduced the number of datasets included and thus, the power of detecting statistically significant differences between the RCR values.

However, the reduction in the number of datasets did not substantially improve the proportion of the variation in the dependant variable explained by the regression model (Table 3). Furthermore, the exclusion of potential confounders also did not yield an overall compliance of sealant retention (as the result of the regression anlalysis for resin sealants shows) with the Prentice criterion either. In addition, the Baker-Kramer graph continued to indicate a difference in slope between the R and G lines (Figure 2), thus suggesting continued non-compliance with the Prentice criterion.

In summary, the sensitivity analysis results indicate limitations of the current evidence and suggest the need for its corroboration through future research on this topic.

### Lack of Compliance with the Prentice Criterion

The current results of this systematic review indicate that the risk of loss of complete retention of sealant materials does not comply with the Prentice criterion. Accordingly, loss of complete retention of sealant materials may therefore not be considered a valid surrogate endpoint for caries occurrence. In clinical practice this means that the caries-preventive efficacy of different sealant materials may not be inferred from the established retention rate, despite its significant correlation, and consequently the suitability of any particular sealant material may not be approved or dismissed on that basis.

Pioneering research in the 1970s demonstrated the caries-preventive effectiveness of placing sealant material on pits and fissures in comparison to no intervention [7]. From these observations the validity of retention rate as surrogate endpoint for caries experience was logically deduced.

However, the logical conjecture that material retention is a valid surrogate endpoint for fissure sealants assumes the ideal situation: that there is only one single pathway from the disease (dental caries) to the true clinical endpoint (carious lesions/cavity development) and that the complete sealant retention in pits and fissures lies on this single pathway. This view does not take into account the possibility that multiple pathways (known and unknown) may exist between the disease and the true clinical endpoint and that the surrogate endpoint may lie on only one of these pathways [12], [14]. In addition, clinical interventions, such as the placement of different sealant materials on pits and fissures, may have different (known and unknown) mechanisms that act independently of a particular surrogate endpoint. Such may be the case with complete sealant retention. Retention may indeed lie on one of the pathways between disease and the clinical endpoint; providing for example, an effective barrier against cariogenic biofilm development in pits and fissures, but not on other known pathways (such as delivery of a lasting anti-cariogenic effect in the tooth enamel through fluoride and mineral release from the sealant material) or indeed, still unknown pathways from the disease to the true clinical endpoint, as Molina et al., 2009 have suggested [29].

Correlation of potential surrogate endpoints with their true clinical endpoints is necessary but not sufficient [12], [15], [30]. Valid surrogate endpoints are obtained on the basis of clinical trials that analyse both the potential surrogate and the true clinical endpoint, in line with suggested methodologies [22]–[24]. It has, moreover, been suggested that surrogate endpoints, if valid, would show strong predictive power, similar to clinical diagnostic tests, regarding their true clinical endpoints, with low numbers of false positive and false negative predictions (not exceeding between 2.5–10.0% of total predictions) [12], [30]. This means that the predictive power of a valid surrogate would be significantly higher than that of random guesses. However, a recent meta-epidemiological study could not establish any caries-predictive power for retention loss of resin-based fissure sealants above that of random guesses: median Diagnostic Odds ratio (25–75% percentile range) 1.21 (0.20–10.71) and 0.28 (0.07–13.10) for resin sealants and random guesses, respectively; Wilcoxon test: z = 0.56; p = 0.58. [31]. As the caries preventive effect of resin-based sealants has been solely attributed to its retention [7], the lack of predictive power of resin-sealant retention gave thus an initial indication (albeit not proof) that sealant retention, in general, may not be compliant with the Prentice criterion for surrogate validity. The results of this systematic review subsequently confirm such lack of compliancy.

Against this background, the use of sealant retention as endpoint in clinical fissure sealant trials, as the quality criterion for the acceptance and rejection of fissure sealant materials and as the basis for guidelines regarding clinical practice may require reconsideration and revision. Complete retention of placed sealants may indeed be relevant as one of many beneficial factors related to the prevention of carious lesion development in dental pits and fissures. However, category errors such as the misinterpretation of ‘beneficial factors’ as ‘valid surrogates’ should be avoided.

### Recommendations for Further Research

The current evidence of this systematic review remains limited and requires corroboration by large sized randomised control trials that compare GIC with resin- based sealants and investigate for both sealant types retention and caries occurrence. Such future trials should avoid attrition bias risk through high loss-to-follow up; include an investigation of performance/detection bias influence into its methodology; test for selection bias using the Berger-Exner test [32] and include all sealed teeth into both retention and caries assessment. It is further recommended that future trials, investigating the clinical efficacy of fissure sealants, should adopt the primary clinical endpoint of caries occurrence (or lack thereof) as study outcome and not only sealant retention rate as surrogate. Potential surrogate endpoints in dentistry should be validiated in line with the Prentice criterion, first, before adopting these as paramount leading endpoints and/or quality criteria in clinical research and daily clinical practice.

## Conclusions

This systematic review has shown that sealant retention does not comply with the Prentice criterion and thus cannot thus be considered a valid surrogate for caries prevention. Against this background, the caries-preventive efficacy of different sealant materials may not be inferred from the established retention rate. Consequently, the suitability of any particular sealant material may not be approved or dismissed on that basis.

## Supporting Information

File S1 **References, extracted data and information from accepted trials.**
(XLS)

File S2 **Assessment of internal validity of trials.**
(XLS)

File S3 **Sensitivity analysis data.**
(XLS)

Checklist S1 **PRISMA 2009.**
(DOC)
